# On the Alteration Which Air and Water Produce on Meat

**Published:** 1811-03

**Authors:** M. Berthollet


### *On the Alteration which Air and Water produce on Meat.* By M. Berthollet.

[Phil. Magazine.]

I BOILED some beef, renewing the water until the liquor no longer gave any precipitation with tannin: I then suspended it in a glass cylinder filled with atmospheric air, and which I placed on a plate filled with water: in a few days the oxygen was changed into carbonic acid; the interior of the cylinder was infected with a putrid smell; the beef that had been boiled gave once more an abundant precipitation with tannin: the ebullition was repeated until the water was no longer disturbed by the tannin: the beef had then almost entirely lost its smell, and it was put again into the apparatus.

The operation was repeated several times, when the following were the results:—

The alteration of the atmospheric air and the disengagement of the putrid smell gradually slackened: the quantity of gelatine formed became progressively smaller: the water upon which the vessel rested gave only feeble indications of ammonia. When I finished the operation there was no longer any putrid smell; but a smell similar to that of cheese, and in fact the animal substance which now preserved scarcely any fibrous appearance, had not only the smell, but precisely the taste of old cheese.

I distilled, separately, an equal weight of beef and of Gruyere cheese, making use of two bell-glasses, each of which communicated with a tube inserted in water; the operation was conducted so as to decompose, as much as possible, the two substances, and to retain all the ammonia which was set free: I compared the quantities of ammonia; that which was furnished by the cheese was to that of the beef nearly in the ratio of 19 to 24: hence it appears that it is one of the distinctive characters of the caseous substance to contain less azote than meat does.

If we may be permitted to draw any inferences from the foregoing imperfect experiments, we may conclude,

1st. That the gelatine which we may obtain from an animal substance is not completely formed in it; but that when this substance has been exhausted by water, it may be once more formed by the action of the air, the oxygen of which is combined with the carbon, while a portion of substance formerly solid becomes gelatinous, as a solid vegetable part becomes soluble by the action of the air.

We must, however, remark, that the property of precipitating with tannin belongs to substances which have very different different properties in other respects: I found that the decoction of Gruyere cheese formed an abundant precipitate with tannin.

2d. That azote enters into the composition of the putrid gas, forming without doubt with hydrogen a combination of an equilibrium less stable than ammonia, or rather an intermediary combination; but when its proportion is diminished to a certain point, it is more strongly retained by the substance, and it ceases to produce putrid gas. This substance, which the putrid odour characterizes, seems rather to be a very evaporable combination, allied to all the gases like the other elastic vapours, than a permanent gas.

3d. Since the caseous part has less azote than most other animal substances, we may conjecture that during life this part is animalized more and more by acquiring a greater proportion of azote and hydrogen: this may be explained by the more intimate combination of oxygen and hydrogen which enter into its composition, and by a separation of the carbon by the act of respiration; so that the last term of chemical action during life has urea for its product, according to the opinion of M. Fourcroy.

